# “Optimizing” the Anesthetic for Cardiac Contractility Modulation Devices

**DOI:** 10.19102/icrm.2025.16032

**Published:** 2025-03-15

**Authors:** Samit Ghia, Ashanay Allen, Ranjit Suri, Himani Bhatt

**Affiliations:** 1 Department of Anesthesiology, Donald and Barbara Zucker School of Medicine at Hofstra/Northwell, Staten Island, NY, USA; 2 Icahn School of Medicine at Mount Sinai, New York, NY, USA; 3 Department of Cardiology, Icahn School of Medicine at Mount Sinai, New York, NY, USA; 4 Department of Anesthesiology, Perioperative & Pain Medicine, Icahn School of Medicine at Mount Sinai, New York, NY, USA

**Keywords:** Cardiac anesthesia, cardiac contractility modulation, electrophysiology, heart failure

## Abstract

We present a case report of a patient undergoing implantation of a cardiac contractility modulation (CCM) device. The Optimizer^®^ Smart Implantable Pulse Generator (Impulse Dynamics, Orangeburg, NY, USA) provides electrical energy to the right ventricular septum to modulate cardiac contractility and improve cardiac function in heart failure patients. The non-excitatory electrical signals are delivered by transvenous leads during the refractory period. Anesthetic administration during implantation can affect appropriate lead positioning. Propofol at high doses can attenuate diaphragmatic contraction and discomfort from inappropriate lead positioning, resulting in diaphragm stimulation. Therefore, local or conscious sedation is preferred during CCM device implantation. In patients undergoing procedures with this CCM device in situ, the Optimizer^®^ activity causes upward deflections of the RS segment on the electrocardiogram. Also, strong electromagnetic fields can disrupt device function. This case report reviews the novel CCM device and its major anesthetic considerations.

## Introduction

The Optimizer^®^ Smart Implantable Pulse Generator (IPG) (Impulse Dynamics, Orangeburg, NY, USA) is a novel cardiac contractility modulation (CCM) device designed to help patients with heart failure.^1^ It is implanted in the right pectoral region, with two leads positioned in the right ventricle. The device delivers impulses to synchronize the electrical activity of the heart, which is shown to increase inotropy. In this case report, we discuss the anesthetic considerations of CCM implantation and patients undergoing procedures with the device in situ.

## Case presentation

A 69-year-old man (height, 1.67 m; weight, 72 kg) with a past medical history significant for coronary artery disease (CAD), ischemic cardiomyopathy (left ventricular ejection fraction [LVEF] of 25%), chronic obstructive pulmonary disease, diabetes mellitus, and peripheral vascular disease presented to the electrophysiology (EP) laboratory for CCM device placement. He underwent multiple percutaneous coronary interventions and dual-chamber implantable cardioverter-defibrillator (ICD) implantation. The patient qualified **(Table 1)** for the implantation of the two-lead Optimizer^®^ Smart IPG device because he continued to have New York Heart Association (NYHA) class II heart failure symptoms despite compliance with guideline-directed medical therapy (GDMT).

**Table 1:** Criteria for CCM Device Implantation

NYHA HF class III or IV Reduced LVEF between 25% and 45% Does not qualify for CRT Refractory to GDMT (eg, sodium–glucose cotransporter-2 inhibitors, β-blockers) QRS duration < 130 ms Normal sinus rhythm

*Abbreviations:* CCM, cardiac contractility modulation; CRT, cardiac resynchronization therapy; GDMT, guideline-directed medical therapy; HF, heart failure; LVEF, left ventricular ejection fraction; NYHA, New York Heart Association.

He presented to the EP laboratory with intravenous access in place. After the placement of a left radial arterial line, anesthesia was induced with midazolam and fentanyl and maintained on a low-dose propofol infusion. During the procedure, he received phenylephrine and dobutamine infusions for hemodynamic support and vancomycin for antibiotic coverage.

After the right pectoral region was prepped and draped in a sterile fashion, the surgical implantation of the Optimizer^®^ Smart IPG began with a 5-cm incision in the infraclavicular region using sharp and blunt dissection to isolate the cephalic branch. A venotomy was performed, and a 0.038″ guidewire was advanced to the inferior vena cava under fluoroscopic guidance. The first ventricular lead was inserted with the assistance of a vein pick and positioned into the mid-right ventricular (RV) septum using a curved stylet. The second ventricular lead was placed through a 6-French peel-away introducer sheath in the subclavian vein over a guidewire and advanced into the right ventricle. Both leads were positioned at least 2 cm apart, avoiding the lateral or anterior free walls of the heart.^2^ The second lead was also actively fixated into the RV septum, approximately 3 cm away from the first lead. The CCM pulse generator was connected to the leads and placed inside the previously created subcutaneous pocket, optimally positioned in a 2.5-cm subcutaneous pocket.^3^ The appropriate location and lead separation were fluoroscopically confirmed.

**Figure 1:**
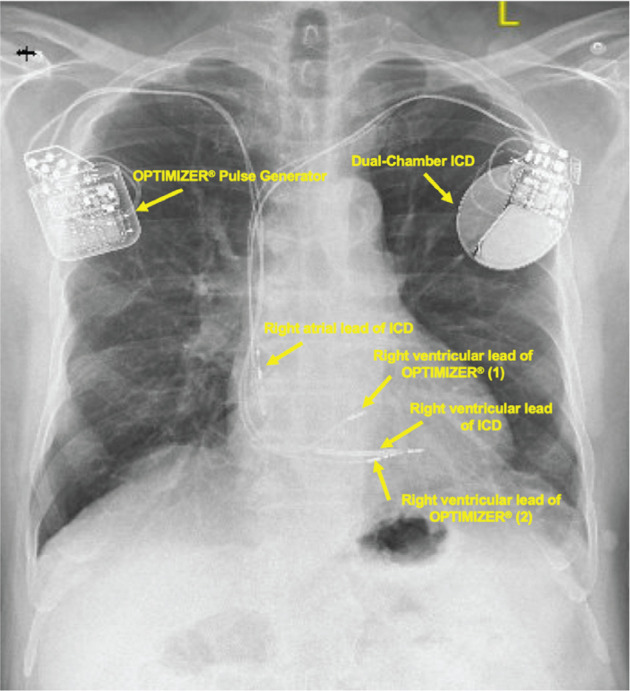
Chest X-ray of the patient with the two-lead OPTIMIZER^®^ Smart Implantable Pulse Generator visible in the upper right pectoral region and a dual-chamber implantable cardioverter-defibrillator visible in the left upper pectoral region. *Abbreviation:* ICD, implantable cardioverter-defibrillator.

Once the device and leads were placed, the patient was awakened to initiate lead placement testing. High-output pacing was performed using standard electrical testing for pacemaker leads to confirm proper placement and functioning and to ensure that the patient did not feel the pacing output. The optimal safety window for CCM therapy and the existing dual-chamber ICD was also tested to ensure that there was no cross-communication.^4^ After proper placement and accurate lead functioning was determined, the Optimizer^®^ was attached to the subcutaneous pectoral fascia using absorbable sutures, and the incision was sealed. The patient’s immediate postoperative course was uncomplicated, and he was discharged the next day. **Figure 1** shows a chest radiograph of the patient with the Optimizer^®^ and an ICD.

Written informed consent was obtained from the patient regarding publishing their data and photographs.

## Discussion

**Figure 2:**
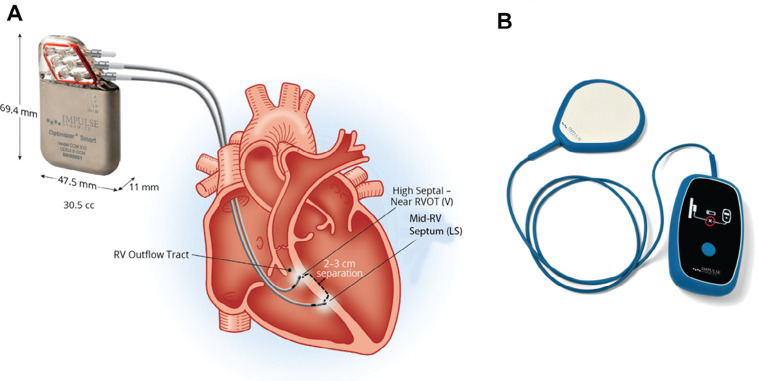
**A:** The OPTIMIZER^®^ Smart Implantable Pulse Generator device with two ventricular leads. **B:** Mobile transcutaneous charger. Image courtesy of Impulse Dynamics. *Abbreviations:* RV, right ventricular; RVOT, right ventricular outflow tract.

**Figure 3:**
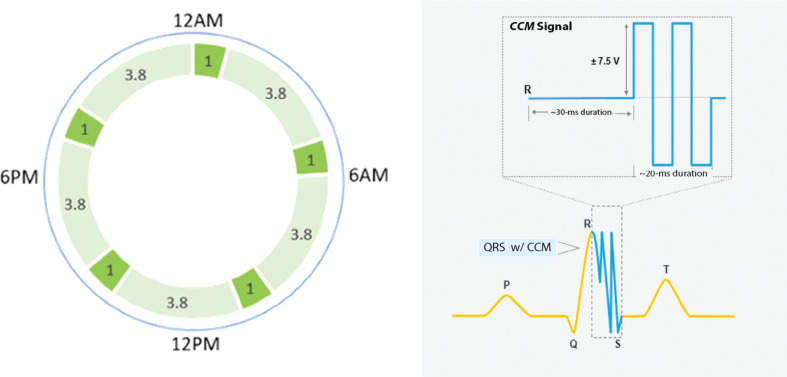
Example of cardiac contractility modulation therapy schedule and the biphasic, non-excitatory, and non-arrhythmogenic signal administered. Image courtesy of Impulse Dynamics. *Abbreviation:* CCM, cardiac contractility modulation.

CCM is a form of heart failure therapy in which a rechargeable device administers biphasic electrical signals to the RV septum during the absolute refractory period of ventricular depolarization with a 30-ms delay before the signal is administered **(Figure 2)**. Therefore, the electrical signals are non-excitatory and non-arrhythmogenic, delivering about 7.5 V of electrical energy over a 24-h period.^1^ The currently approved therapy schedule is five 1-h therapy sessions, as seen in **Figure 3**. The Optimizer^®^ also uses an at-home transcutaneous charger that only requires charging once a week for approximately 60 min **(Figure 2)**.^5^

The device has a sensor within the two leads that senses local cardiac activity. It uses an algorithm that incorporates the sensed local cardiac activity, time between R–R intervals, and signal detection at irregular intervals to generate an appropriate CCM signal.^1,6^ Once all criteria are met and the signal is generated, the device continues to monitor the heart in anticipation of the next signal. The leads also detect arrhythmogenic activity that interferes with appropriate contractions (ie, premature ventricular contractions, ventricular tachycardia, etc.). If such activity is detected, the device forgoes therapy delivery until the next regular heartbeat.^7^ Finally, if a cardiac implantable electronic device (CIED) is present, the pulse generator can be programmed to coordinate with the device and only initiate therapy during specific intervals (eg, the refractory period of a stimulated cardiac contraction).

Several studies have examined the specific mechanism of action of CCM technology and concluded that it occurs through multiple avenues of calcium upregulation,^8^ which include L-type calcium channels, sarcoplasmic/endoplasmic reticulum Ca^2+^-ATPase 2a activity, and phosphorylation of phospholamban.^8^ This was confirmed when antagonism of calcium channels and upregulation resulted in an attenuated effect of CCM.

Another sign of CCM’s benefit is the reversal of chronic heart failure biomarkers. For example, in patients with NYHA class III heart failure, cardiomyocytes express fetal and stretch response genes, atrial natriuretic peptides, and B-type natriuretic peptides.^9^ However, after treatment with CCM, these markers were significantly reduced. When compared to patients with standard heart failure medical management, patients with CCM technology had significantly improved contractility, cardiomyocyte structure, LVEF, and stroke volume.^10^ Ultimately, CCM technology appears to decrease myocardial stress, allowing for improvements in cardiac function.

When the device delivers its initial electrical signal, it can increase trabeculae contractility by 50%–70%, confirming that CCM therapy is highly advantageous for qualified heart failure patients.^11^ Previous studies on CCM therapy have shown significant improvements in septal contractions, decreased heart failure-related hospitalizations, and decreased cardiac mortality. Additionally, the device enhanced the 6-min hall walk distance and quality of life.^12–14^

### Indications

The Optimizer^®^ Smart IPG is an approved CCM device in a specific subset of heart failure patients, which uses two ventricular leads to deliver electrical therapy. Patients qualifying for this device are those who are classified as NYHA functional class III or IV, have a QRS duration of <130 ms, and have an LVEF between 25% and 45%. These candidates remain symptomatic despite GDMT.^15^ Furthermore, they are not suitable for cardiac resynchronization therapy or left ventricular assist devices. **Table 1** summarizes the criteria to qualify for CCM device implantation. The two-lead Optimizer^®^ Smart IPG is contraindicated in patients with mechanical tricuspid valves and poor vascular access that is prohibitive to implantation.^1^

### Surgical complications

Once the device and leads are placed, the ventricular leads are tested using standard electrical testing for pacemaker leads to confirm proper placement and functioning. Improper device or lead placement (eg, subcutaneous pocket is too superficial or electrode is placed near the anterior free wall) can result in chest wall discomfort, skin erosion, electrical stimulation of skeletal muscle, varying output strengths from the ventricular leads, and/or no improvement in heart failure.^13^ Furthermore, improper device placement can lead to more serious complications, such as venous thrombosis, perforation of the ventricular wall, and vascular injury to the subclavian vein.^16^ Thus, adequate surgical planning and preoperative consideration prior to implantation can help to mitigate such complications.

### Anesthetic considerations for implantation

Patients receiving the Optimizer^®^ Smart IPG have moderate-to-severe heart failure and likely other comorbidities (eg, CAD, hypertension, venous thromboembolism), which can require more comprehensive anesthetic management.^17^ A thorough preoperative assessment must investigate their comorbidities, medication history, physical findings, and diagnostic tests. A transthoracic echocardiography will identify cardiac function, while a chest X-ray can confirm the presence of a CIED. If present, the anesthesiologist should confirm that it is disabled prior to incision. Lastly, the anesthesiologist must verify that any prescribed anticoagulants are appropriately held.

The current recommendation from the device manufacturer is to closely monitor patients’ ventricular rhythm during lead implantation and activation.^1^ The decision of which monitoring techniques to use rests with the anesthesiology team and can depend on patient comorbidities and possible complications. According to Kuschyk et al., non-invasive cardiac output monitors, echocardiography, and even continuous beat-to-beat monitors are equally sufficient to use during implantation to monitor sudden hemodynamic shifts in patients.^18^ Intraoperative echocardiography monitoring can provide comprehensive data regarding cardiac structure and function, cardiac output, and rapid identification of potential complications.^19^ However, appropriate monitoring during device implantation is dependent upon patient comorbidities, vasopressor and inotropic requirements, and complications expected during device placement. Some patients with significant comorbidities may require invasive monitoring with an arterial line and/or central venous access. This allows for more acute and precise monitoring of changes in blood pressure and prompt administration of vasoactive medications or fluid management.

During implantation, local anesthetic or conscious sedation (eg, midazolam and fentanyl) is preferred because these drugs permit patient communication during lead testing to confirm appropriate lead placement.^18^ Propofol, on the contrary, can depress diaphragmatic contraction and sensation, and higher doses can obscure lead testing.^20^ However, current literature and CCM device implantation recommendations do not preclude the use of propofol. They do state that sedation should be administered at the lowest possible dose and propofol administration should be isolated to patients who cannot tolerate the procedure at the manufacturer-recommended lower levels of sedation.^18^ Higher levels of sedation may be required during the creation of the subcutaneous pocket for the placement of the pulse generator, which is a more stimulating step in the procedure. One solution to propofol-induced interference of lead testing is to test prior to creating the pocket. After lead testing is completed under conscious sedation, propofol can be titrated to patient comfort during the more stimulating parts of the procedure. The timing of the pocket creation is based on the preference of the perioperative team and their patient concerns (eg, minimal anesthesia use, blood loss management).^18^

Following successful implantation of the device, standard postoperative care is necessary for at least 24 h to rule out surgical complications like lead fracture, pneumothorax, inappropriate lead placement causing diaphragmatic stimulation, and uncomfortable thoracic sensations or perforation.^18,21^ An echocardiography, a chest X-ray, and device interrogation are necessary to verify proper placement and functionality.^22^ Patients with CIEDs will require additional testing to determine appropriate functioning of both devices. In addition, patients will receive device education, which should review operating, charging, and environmental cautions (eg, electromagnetic fields).

### Anesthetic considerations after implantation

As advancements in heart failure technology continue and devices such as the Optimizer^®^ Smart IPG become more widely implemented, it is crucial for anesthesiologists to understand the anesthetic implications of caring for patients with these devices in future surgeries. In these cases, standard preoperative anesthetic management—detailed patient history, thorough physical examination, assessment of cardiac function, and pertinent diagnostic workup—should be completed.

**Figure 4:**
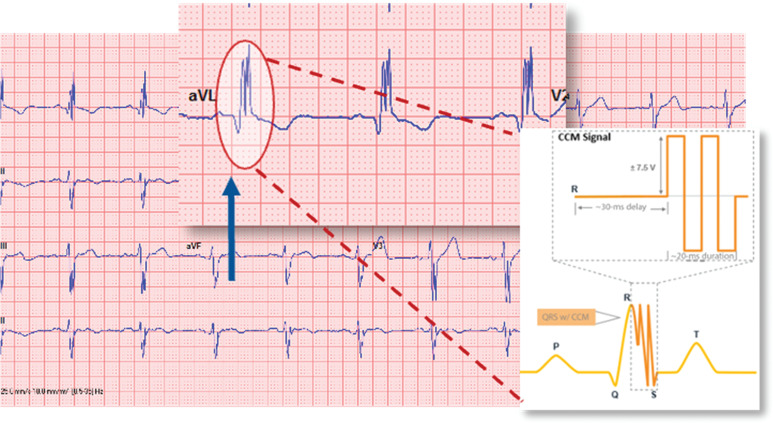
Example electrocardiogram of biphasic cardiac contractility modulation therapy delivery. Image courtesy of Impulse Dynamics. *Abbreviation:* CCM, cardiac contractility modulation.

If a patient with the Optimizer^®^ Smart IPG presents for a surgical procedure, anesthesiologists should be aware that their electrocardiogram (ECG) morphology can be distorted. As noted earlier, CCM technology uses biphasic voltages 30 ms after detection of local cardiac activity during the absolute refractory period, with a total signal duration of about 20 ms. Therefore, on an ECG, the RS segment of the QRS complex will present with multiple upward spikes, changing the appearance of the complex **(Figure 4)**.^23^

If external cardioversion or defibrillation is necessary, defibrillation paddles should not be placed close to or directly above the device, as this could result in dysfunction of the leads or force the device into standby. Following defibrillation, the device should be interrogated for proper function and therapy delivery. Caution should be taken when exposing patients to strong electromagnetic fields or procedures involving high-frequency electrical currents (ie, nuclear magnetic resonance, magnetic resonance imaging [MRI], radiofrequency ablation, electrocautery, or therapeutic radiation).^1^ The device is magnetic resonance conditional (1.5-T MRI environment) and could become inactivated if specific criteria are not met. However, if a patient requires a procedure with high-frequency electrical currents, the device should either be shielded from the radiation or deactivated, and the patient’s blood pressure should be closely monitored. When the device is reactivated, device function should be assessed.^6^

## Conclusion

The Optimizer^®^ Smart IPG has shed light on the promising role of CCM technology to improve the quality of life for individuals with moderate-to-severe heart failure. This case report highlighted the implantation and anesthetic sequelae of this CCM device. As heart failure devices become more widely used, it is imperative that we recognize the unique anesthetic management and implant considerations for these patients. **Table 2** summarizes the major takeaways from this case report.

**Table 2:** CCM Technology Major Takeaways

Indications	NYHA HF class III or IV Ineligible for CRT Reduced LVEF between 25% and 45% QRS duration < 130 ms Normal sinus rhythm
CCM technology	Non-excitatory, biphasic electrical signals Administered during the absolute refractory period
Surgical implantation	Implanted in the upper right pectoral region Two leads positioned in the RV septal wall
Anesthetic considerations for implantation	Prefer local anesthetic or conscious sedation Awaken patient upon testing Avoid deep sedation
Anesthetic considerations after implantation	ECGs may present with multiple upward spikes on the QRS complex Caution when exposing patients with this device to strong electromagnetic fields

*Abbreviations:* CCM, cardiac contractility modulation; CRT, cardiac resynchronization therapy; ECG, electrocardiogram; HF, heart failure; LVEF, left ventricular ejection fraction; NYHA, New York Heart Association; RV, right ventricular.
